# Phosphine inhibits transcription of the catalase gene through the DRE/DREF system in *Drosophila melanogaster*

**DOI:** 10.1038/s41598-017-13439-4

**Published:** 2017-10-10

**Authors:** Tao Liu, Li Li, Baishu Li, Guoping Zhan

**Affiliations:** Institute of Equipment Technology, Chinese Academy of Inspection and Quarantine, No. 241, Huixinxijie, Chaoyang District, Beijing, 100029 P.R. China

## Abstract

Phosphine (PH_3_) is a toxin commonly used for pest control. Its toxicity is attributed primarily to its ability to induce oxidative damage. Our previous work showed that phosphine could disrupt the cell antioxidant defence system by inhibiting expression of the catalase gene in *Drosophila melanogaster* (*DmCAT*). However, the exact mechanism of this inhibition remains unclear. Here, we implemented a luciferase reporter assay driven by the *DmCAT* promoter in *D*. *melanogaster* S2 cells and showed that this reporter could be inhibited by phosphine treatment. A minimal fragment of the promoter (−94 to 0 bp), which contained a DNA replication-related element (DRE) consensus motif (−78 to −85 bp), was sufficient for phosphine-mediated reporter inhibition, suggesting the involvement of the transcription factor DREF. Furthermore, phosphine treatment led to a reduction in *DREF* expression and consequent repression of *DmCAT* transcription. Our results provide new insights on the molecular mechanism of phosphine-mediated catalase inhibition. Phosphine treatment leads to reduced levels of the transcription factor DREF, a positive regulator of the *DmCAT* gene, thereby resulting in the repression of *DmCAT* at transcriptional level.

## Introduction

Phosphine (PH_3_) has been widely used as an insecticide for grain reserves since the 1930s. Due to various advantages, including its low cost, high toxicity, minimal residue, and rapid action, PH_3_ has replaced the ozone-harming chemical methyl bromide as the most popular fumigant worldwide^1^. However, with prolonged use of PH_3_, pests with high levels of resistance have been reported in many countries, such as the United States, Australia, and Brazil^2–4^. As there is no effective chemical replacement for PH_3_, much effort has been devoted to studying the mechanisms of PH_3_ toxicity and resistance^5^.

To date, the precise mode of PH_3_ toxicity is still unclear. One of the mechanisms involved is the generation of reactive oxygen species (ROS)^6^. ROS can lead to oxidative stress, lipid peroxidation, and toxic changes to the redox state of the cell, leading to a failure of oxidative respiration^7–10^. In support of this, antioxidants such as glutathione and melatonin can prevent most of the oxidative damage induced by PH_3_^11,12^.

PH_3_ can also disrupt the antioxidant defence system^13^. Typically, high levels of ROS will activate the antioxidant defence system of the cell^14^, inducing the up-regulation of various antioxidant enzymes such as superoxide dismutase (SOD) and catalase (CAT). However, while SOD is up-regulated after PH_3_ treatment, expression and activity of CAT, which can decompose hydrogen peroxide to water and oxygen, are inhibited by PH_3_^15,16^. It was proposed that PH_3_ may inhibit CAT by reducing the metal ion cofactor in the active site^17^. However, the inhibitory effect could only be observed *in vivo* but not *in vitro*, suggesting that there may be other regulatory mechanisms^16,18^. Previously, we found that PH_3_ fumigation could reduce mRNA production of the *DmCAT* gene in *Drosophila melanogaster*, which was the first evidence that PH_3_ may disrupt the antioxidant defence system at transcriptional level, adding another layer of complexity to the function of PH_3_^19^. However, the mechanism by which PH_3_ inhibits *DmCAT* transcription is unknown.

In the current study, we investigated whether PH_3_ could directly regulate the transcription of *DmCAT* by dissecting the role of the *DmCAT* promoter in PH_3_-mediated *DmCAT* inhibition in *D*. *melanogaster* S2 cells. We first established a PH_3_ treatment system using S2 cells and confirmed that it led to down-regulation of *DmCAT*. We then developed a luciferase reporter assay and identified an essential fragment containing a DNA replication-related element (DRE) within the *DmCAT* promoter. This element was sufficient and necessary for PH_3_-mediated *DmCAT* repression. We then showed that levels of DREF, the DRE-binding transcription factor, were reduced upon PH_3_ treatment and that this phenomenon was an essential prerequisite for *DmCAT* repression. These data suggest that PH_3_ inhibits *DmCAT* promoter activity by reducing levels of the transcription factor DREF, providing a new mechanism for the mode of action of PH_3_.

## Results

### PH_3_ treatment reduces the expression of *DmCAT* in *D*. *melanogaster* S2 cells

**Figure 1:**
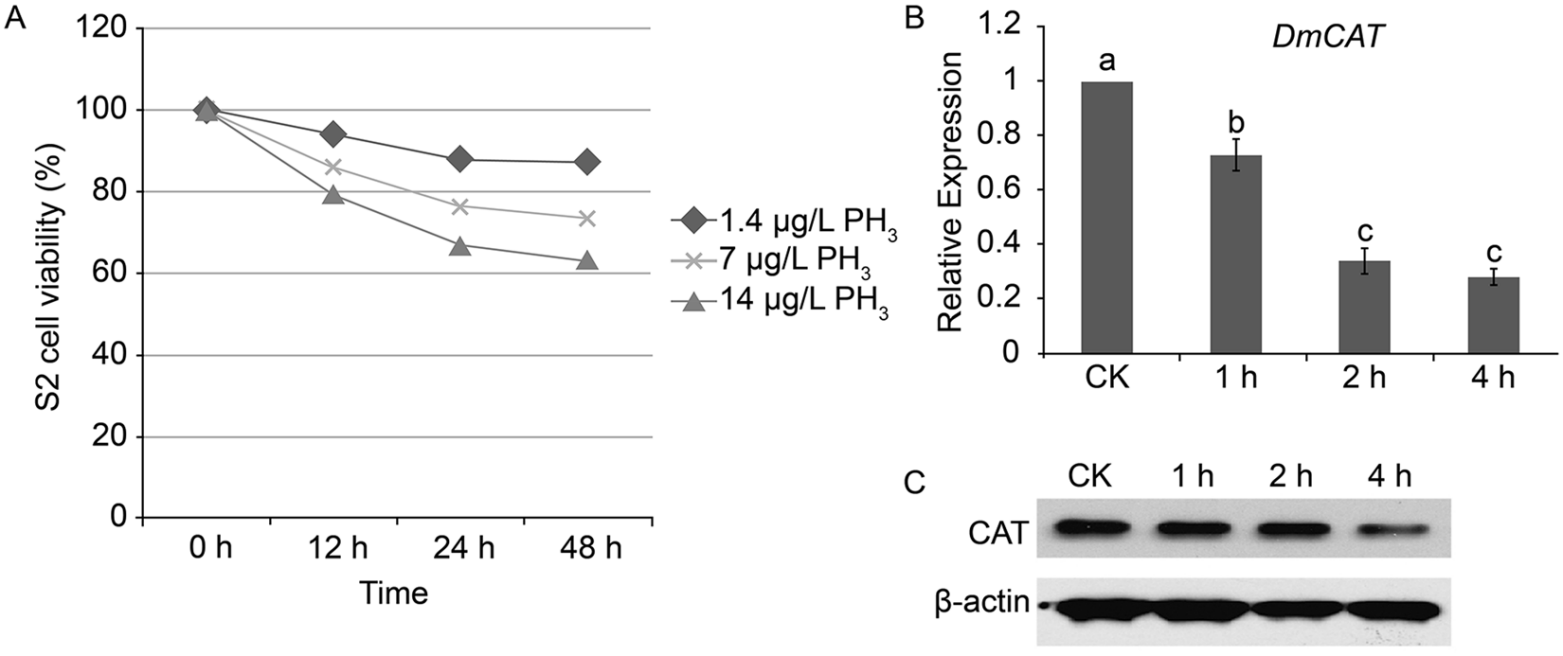
Establishment of phosphine (PH_3_) treatment system in *Drosophila melanogaster* S2 cells. (**A**) Cell viability assays for S2 cells treated with different concentrations of PH_3_ for various lengths of time. (**B**) Real-time PCR analysis of the *DmCAT* gene in S2 cells treated with 14 µg/L PH_3_ for 0, 1, 2, or 4 h. CK: control. Lowercase letters indicate significant differences at p < 0.05. (**C**) Western blot against DmCAT in S2 cells treated with 14 µg/L PH_3_ for 0, 1, 2, or 4 h (Full-length blots are presented in Supplementary Figs S1 and S2).

To gain insight into the molecular mechanism of PH_3_-mediated *CAT* gene repression, we established a PH_3_ treatment system in *D*. *melanogaster* S2 cells. We observed that PH_3_ treatment inhibited the growth of S2 cells, with clear dose and time dependencies (Fig. 1A). At a concentration of 14 µg/L and a treatment time of 48 h, PH_3_ led to the death of 40% of cells.

To examine the expression of the *DmCAT* gene, we performed reverse transcription real-time PCR and western blotting after treating S2 cells with 14 µg/L PH_3_ for 1, 2, and 4 h. We observed a considerable decrease in *DmCAT* at both mRNA and protein level (Fig. 1B,C). These data indicated that this system could faithfully recapitulate PH_3_-mediated growth inhibition and *CAT* gene repression^19^.

### A DRE consensus motif within the *DmCAT* promoter is required for PH_3_-mediated transcription inhibition

**Figure 2:**
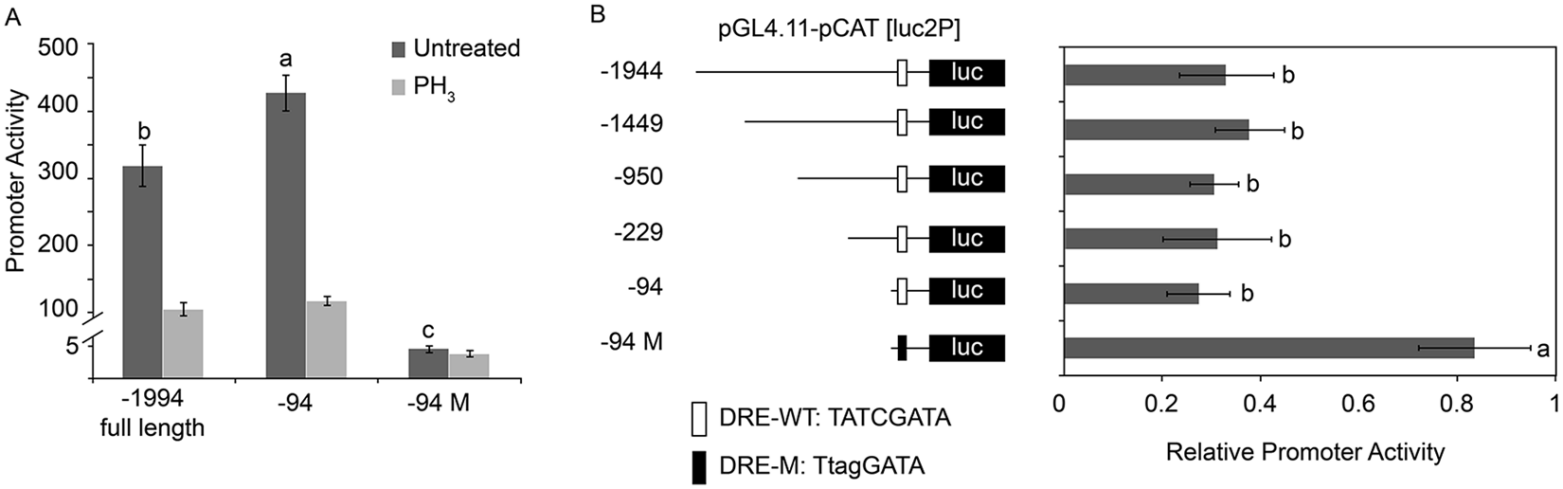
A minimal fragment containing a DNA replication-related element (DRE) motif is required for phosphine (PH_3_)-mediated *DmCAT* repression. (**A**) Promoter activity of wild-type (−1,944 full length and −94) and mutant (−94 M) DRE *DmCAT* promoters treated with or without PH_3_. (**B**) Luciferase assay comparing PH_3_ inhibition efficiencies of full-length (−1,944) and various truncated fragments of *DmCAT* promoter. Relative promoter activity was defined as the ratio of luciferase activity in PH_3_-treated cells to that in untreated cells. DRE-WT, wild-type DRE; DRE-M, mutant DRE. Lowercase letters indicate significant differences at p < 0.05.

To directly investigate the role of the *DmCAT* promoter in PH_3_-mediated *DmCAT* repression, we cloned the full-length (−1,944 to 0 bp)^20^ and various truncated fragments of the *DmCAT* promoter into the pGL4.11 [luc2P] luciferase reporter vector. PH_3_ treatment reduced luciferase activity to 32.9% when the full-length *DmCAT* promoter was used, indicating that the promoter was directly involved in PH_3_-mediated *DmCAT* repression (Fig. 2A). However, four truncated promoter fragments showed similar efficacy in reducing luciferase activity upon PH_3_ treatment (Fig. 2B), suggesting that the core response unit lay within the −94 to 0 bp region.

It was previously reported that a DRE element was present in the *DmCAT* promoter at positions −78 to −85 bp^20^. This element, together with its binding protein, DREF, is required for the positive regulation of *DmCAT* during fruit fly development^20^. To investigate whether this DRE element was required for PH_3_-mediated *DmCAT* repression, we mutated three critical nucleotides within the DRE consensus sequence and assessed the mutant promoter with the luciferase assay. Consistent with a positive role of DRE in *DmCAT* regulation, DRE mutations strongly reduced promoter activity (by 99%) in S2 cells without PH_3_ treatment (Fig. 2A). Interestingly, after treatment with PH_3_, relative luciferase activity was reduced by only 16.5% when using the DRE mutant promoter rather than by 61.7%, as observed for the full-length promoter (Fig. 2A,B and Supplementary Table S1). These data suggested that DRE was critical for PH_3_-mediated *DmCAT* repression.

### PH_3_ reduces the activity of the *DmCAT* promoter through inhibition of *DREF* expression

**Figure 3:**
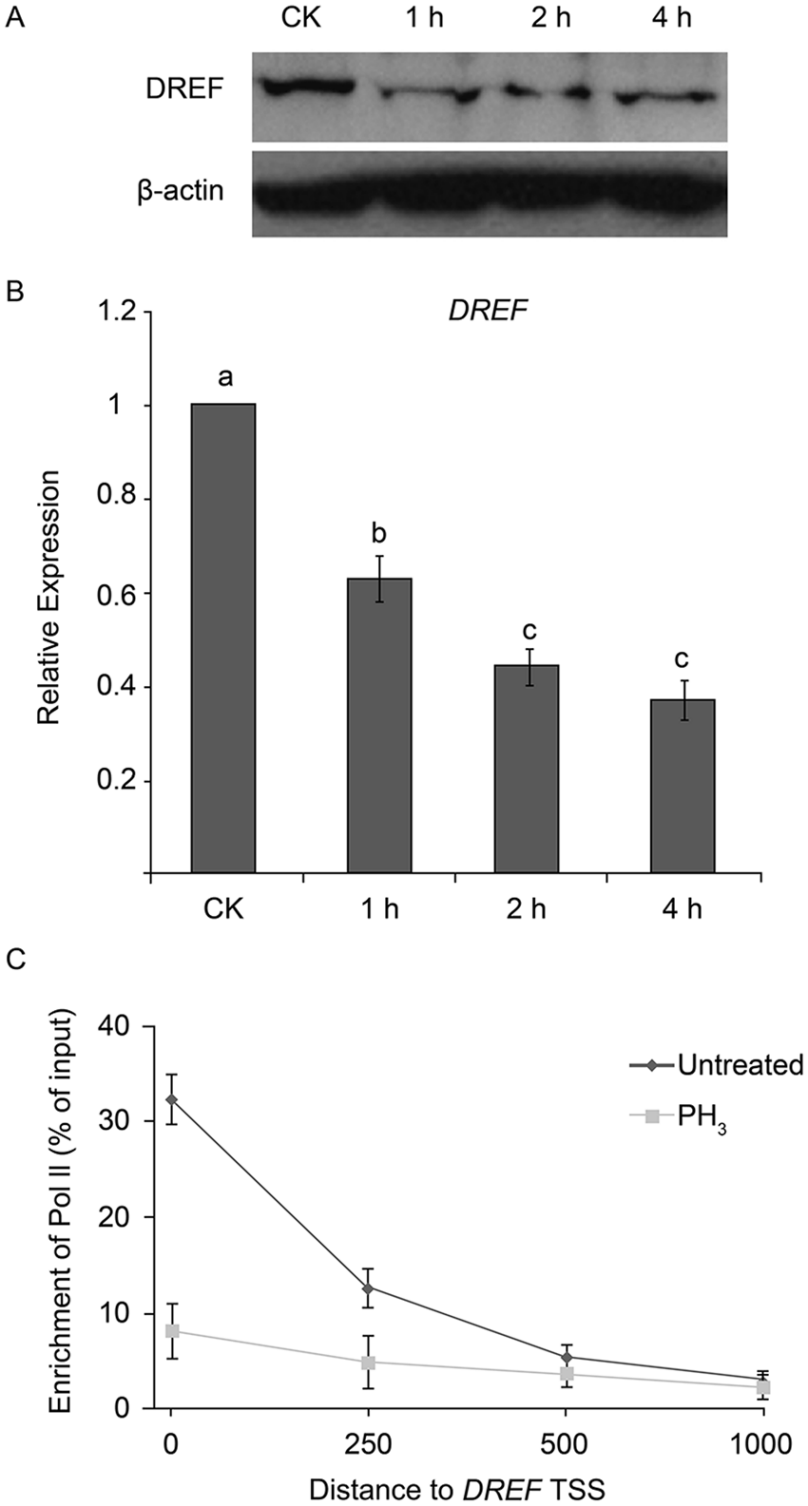
Repression of DREF by phosphine (PH_3_). (**A**) Western blot (Full-length blots are presented in Supplementary Figs S3 and S4) and (**B**) real-time PCR analysis of *DREF* in S2 cells treated with 14 µg/L PH_3_ for 0, 1, 2, or 4 h. CK: control. (**C**) Chromatin immunoprecipitation (ChIP)-qPCR analysis of Pol II enrichment on the *DREF* promoter with or without PH_3_ treatment. Lowercase letters indicate significant differences at p < 0.05. TSS, transcription start site.

Previously, we found that PH_3_ treatment led to reduced DREF levels in the fruit fly^19^. We observed similar effects in S2 cells at both protein and mRNA level (Fig. 3A,B). Additionally, a Chip-qPCR analysis was performed to compare the occupancy of Pol II on the *DREF* promoter with or without PH_3_ treatment. Our results showed that Pol II occupancy was greatly reduced upon PH_3_ treatment, suggesting that transcription activity could directly contribute to reduced *DREF* mRNA abundance (Fig. 3C). These data provided a possible explanation for PH_3_-mediated *DmCAT* repression, with PH_3_ down-regulating the expression of *DREF* at transcriptional level, and low amounts of DREF resulting in reduced *DmCAT* promoter activity.

**Figure 4:**
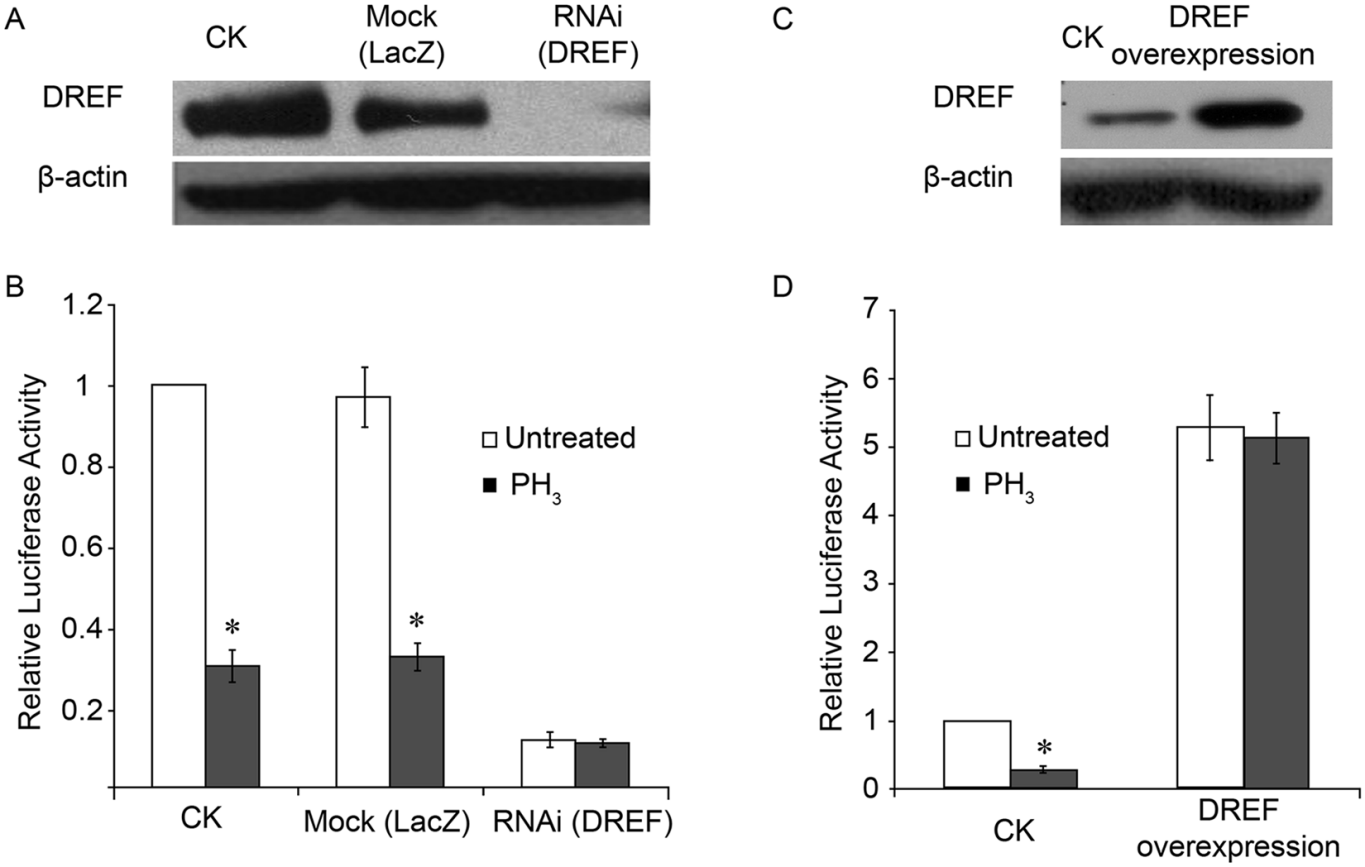
Effect of *DREF* RNAi and overexpression on phosphine (PH_3_)-mediated *DmCAT* repression. (**A**) Representative western blot validating the efficiency of *DREF* RNAi. CK: control (Full-length blots are presented in Supplementary Figs S4 and S5). (**B**) Luciferase assay comparing relative luciferase activities of control and *DREF* RNAi cells with or without PH_3_ treatment. *p < 0.05 vs. untreated cells. (**C**) Representative western blot validating the overexpression of DREF (Full-length blots are presented in Supplementary Figs S4 and S6). (**D**) Luciferase assay comparing relative luciferase activities of control or DREF overexpressing cells with or without PH_3_ treatment. *p < 0.05 vs. untreated cells.

To test this hypothesis, we performed the luciferase assay under *DREF* knockdown or overexpression conditions. In both cases, we would expect luciferase activity not to respond to PH_3_ treatment, since there would be either no DREF or constitutively high levels of DREF, respectively. Double-stranded RNA-mediated *DREF* knockdown efficiently depleted DREF protein levels (Fig. 4A). Consistent with the positive role of the DRE/DREF system in *DmCAT* regulation, we observed reduced luciferase activity after *DREF* knockdown (Fig. 4B). After PH_3_ treatment, we observed a dramatic decrease in luciferase activity in mock knockdown cells but not in *DREF* knockdown cells, suggesting that DREF is essential for PH_3_-mediated *DmCAT* repression (Fig. 4B).

For DREF overexpression, we cloned the full-length *DREF* open reading frame into the pAc5.1/V5-His expression vector and transfected this vector along with the pGL4.11-pCAT [luc2P] luciferase reporter. Western blot confirmed the overexpression of DREF protein (Fig. 4C). We observed higher luciferase activity in *DREF* overexpressing cells, again supporting its positive role in *DmCAT* expression. After PH_3_ treatment, we found that luciferase activity was reduced only in control transfected cells and not in *DREF* overexpressing cells, further supporting our hypothesis (Fig. 4D).

### Effect of PH_3_ on the expression of DREF target genes

**Figure 5:**
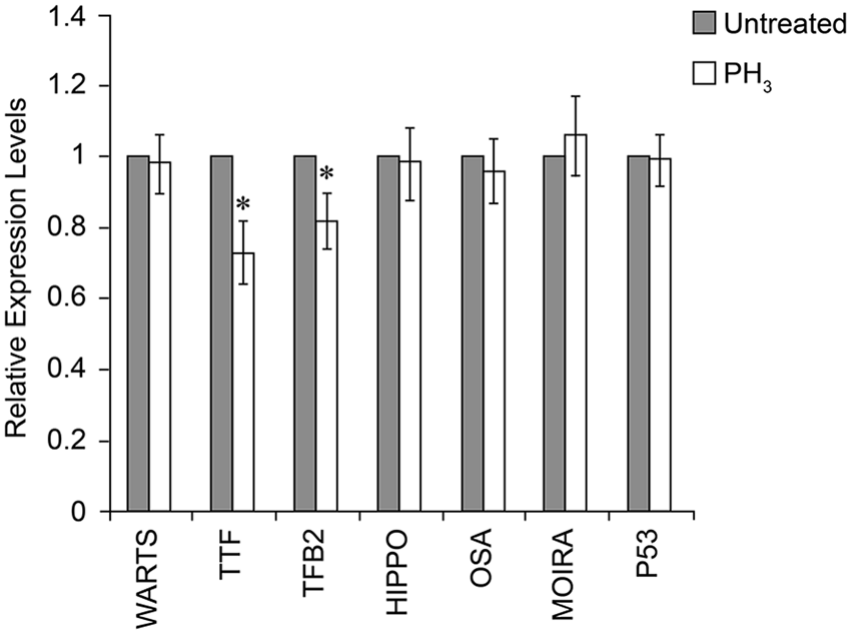
Effect of phosphine (PH_3_) on the expression of seven DREF target genes. *p < 0.05 vs. untreated cells.

As recently reviewed by Nguyen *et al*., DREF is a multifunctional factor, and could positively regulate the expression of genes other than *CAT* ^21^. To determine whether these DREF target genes were also targets of PH_3_, we investigated the expression of seven DREF target genes including *WARTS* ^22^, *TTF* ^23^, *TFB2* ^24^, *HIPPO* ^25^, *OSA* ^26^, *MOIRA* ^26^, and *P53* ^27^ in S2 cells after PH_3_ treatment. Our results showed that the expression of *TTF* and *TFB2*, which are related to mitochondrial function, were partly reduced after PH_3_ treatment, indicating that the DRE/DREF system might play a common role in relaying the PH_3_ signal to its target genes (Fig. 5).

## Discussion and Conclusion

This and our most recent studies demonstrate that PH_3_ inhibits catalase activity via transcriptional regulation^19^. Previous studies mostly focused on the direct inhibition of catalase enzymatic activity by PH_3_^15,16^. While these two mechanisms are not necessarily mutually exclusive, it should be noted that the enzymatic inhibition of catalase could only be observed *in vivo* but not *in vitro*, suggesting that enzymatic regulation might not be direct^16,18^. In addition, reduction of the metal ion cofactor of catalase by PH_3_ could not explain why PH_3_ has the opposite effect on superoxide dismutase, which also has metal cofactors.

The observation that PH_3_ treatment results in down-regulation of *DmCAT* mRNA in *D*. *melanogaster* led us to investigate how PH_3_ affected *DmCAT* transcription at the molecular level. By devising a luciferase reporter system in *D*. *melanogaster* S2 cells, we demonstrated that *DmCAT* promoter activity was strongly repressed upon PH_3_ treatment. This response was quick (within 1 h), indicating that it may not be a downstream effect of antioxidant system failure. Further dissection of the *DmCAT* promoter showed that a small fragment (−94 to 0 bp) was necessary and sufficient for PH_3_ responsiveness (Fig. 2). Interestingly, we found that a DRE motif (−78 to −85 bp) was involved in this process. The *DmCAT* promoter with a mutated DRE sequence no longer responded to PH_3_ treatment, although basal promoter activity was greatly reduced. It has been reported that the *CAT* gene is positively regulated by the DRE/DREF system in *D*. *melanogaster* ^20^, which raises an interesting question as to why a positive regulatory element would be involved in the negative regulation of the *CAT* gene.

The finding that levels of the positive regulatory factor DREF decrease after PH_3_ treatment provides a reasonable explanation for this discrepancy. PH_3_ treatment does not directly inhibit the *DmCAT* promoter, but rather it reduces levels of the positive regulatory factor DREF. By manipulating levels of DREF via RNAi or overexpression experiments, we report that (1) the DREF level positively correlates with *DmCAT* promoter activity, consistent with a previous study^20^; and (2) either depleting or overexpressing *DREF* renders the *DmCAT* promoter nonresponsive to PH_3_ treatment, suggesting that DREF is the critical factor for PH_3_-mediated *DmCAT* repression. Thus, the down-regulation of DREF by PH_3_ treatment is a prerequisite for repression of *DmCAT*.

DRE and the DRE-binding factor DREF are recognised as a multifunctional system with important roles in *D*. *melanogaster*, including tumour suppression, cell development, tissue growth, chromatin organisation, and mitochondrial biogenesis^21^. Therefore, it is interesting to know whether other DRE/DREF target genes are also down-regulated by PH_3_ and, if so, what are their roles in PH_3_ toxicity. Our results showed that two DREF target genes related to mitochondrial function were down-regulated after PH_3_ treatment (Fig. 5). Considering that PH_3_ could also disrupt mitochondrial function and inhibit respiration in invertebrate organisms^6^, it is possible that the DRE/DREF system is also involved in this process and is essential for relaying the PH_3_ signal to its targets genes. This will require comprehensive analysis of transcriptional changes upon PH_3_ treatment. Additional questions, which will be explored in future studies, include the exact down-regulation of DREF by PH_3_.

Investigations into how PH_3_ disrupts the antioxidant defence system and leads to the generation of ROS have produced important results in the field, especially in the context of emerging PH_3_ resistance. First reported more than two decades ago, PH_3_ resistance has now been observed in many countries. It may become a worldwide problem in the future, considering its predominant use and the consequent likelihood of imposing strong selection for PH_3_ resistance in pests. Studies on the molecular mechanism of PH_3_ toxicity will provide information to help address PH_3_ resistance, ultimately benefitting industries that rely on pest control.

## Materials and Methods

### Preparation of the PH_3_ solution

Gaseous PH_3_ was generated by adding aluminium phosphide tablets to acidified water^28^. To make the PH_3_ solution, PH_3_ gas (1 mL) was injected into a sealed, sterile Wheaton narrow-mouth bottle (Z250007; Sigma, St. Louis, MO, USA) containing 1 × PBS, pH 7.4 (15 mL), through a Mininert Valve (33304; Sigma). The bottle was inverted overnight at 28 °C to allow it to equilibrate. The concentration of the PH_3_ solution was determined to be 14 µg/mL using a QuantiChrom Phosphate Assay Kit (BioAssay Systems, Hayward, CA, USA) according to the manufacturer’s protocol.

### S2 cell culture and PH_3_ treatment procedures

*D*. *melanogaster* S2 cells were cultured in TC100 medium supplemented with heat-inactivated FBS (10%) and antibiotics at 28 °C. For cell viability assays, the cells were seeded into a 96-well plate at a density of 0.5 × 10^5^ per well. After 24 h, the medium was changed to fresh medium, and cells were treated with PH_3_ at a concentration of 1.4, 7, or 14 µg/L for 0, 12, 24, or 48 h. At each time point and for each treatment concentration, cell viability was evaluated using the CellTiter 96^®^ AQ_ueous_ One Solution Cell Proliferation Assay System (Promega, Madison, WI, USA)^29^.

For real-time PCR and western blot assays, S2 cells were seeded into a 6-well plate at a density of 5 × 10^5^ per well. Cells were treated with PH_3_ at a concentration of 14 µg/L for 1, 2, or 4 h, and then were harvested for RNA and protein extraction.

### Luciferase reporter construction and luciferase assay

There is only one identified gene encoding for CAT in *D*. *melanogaster*. The full-length promoter of *DmCAT* (−1,944 to 0 bp, relative to the transcriptional start site of *DmCAT*) was amplified with the following two primers: Full-Pcat-S-*XhoI*, 5′-CTCGAGACCTGGGTTTATGGGCTAA-3′; Full-Pcat-A-*BglII*, 5′-AGATCTGTAGTCAATCAACTGATTGGA-3′^20^. Differently truncated fragments of the *DmCAT* promoter were amplified with the forward primers shown in Table 1, and Full-Pcat-A-*BglII* was used as the reverse primer. To mutate the DRE sequence, which corresponds to positions −85 to −78 bp, the forward primer −94M-Pcat-S-*XhoI*, carrying three nucleotide mutations, was used.

**Table 1:** Primers for luciferase reporter construction.

Primer name	Sequence 5′–3′
−1,449-Pcat-S-*XhoI*	CTCGAGCAATTGGCTTCATGTTTCGTTTTCTTG
−950-Pcat-S-*XhoI*	CTCGAGAGTCAAGATTCAGACAATGTGCCTAC
−229-Pcat-S-*XhoI*	CTCGAGATCTTAATGGTGTTGGGACA
−94-Pcat-S-*XhoI*	CTCGAGTAATCGAAATATCGATATCTTCGGC
−94M-Pcat-S-*XhoI*	CTCGAGTAATCGAAATtagGATATCTTCGGC^1^

^1^Mutant nucleotides are denoted in lowercase.

The amplified fragments were cloned into the pGL4.11 [luc2P] vector with *XhoI* and *BglII*, resulting in pGL4.11-pCAT [luc2P]. This reporter, together with the control vector pGL4.74 [hRluc/TK], was co-transfected into S2 cells for 48 h^30^. S2 cells were then treated with PH_3_ for another 4 h, followed by detection of luciferase activity with the Dual-Luciferase Reporter Assay System (Promega).

### Real-time PCR

Total RNA was extracted using TRIzol reagent (Invitrogen, Carlsbad, CA, USA) according to the manufacturer’s instructions. Contaminating genomic DNA was removed using DNase I (Takara, Kusatsu, Japan). RNA (2 µg) was reverse transcribed using M-MLV Reverse Transcriptase (Promega). Real-time PCR was carried out with SYBR Green Real-time PCR master mix (Toyobo, Osaka, Japan). *RP49* was used as an endogenous control. The primers used are listed in Table 2.

**Table 2:** Primers for real-time PCR.

Gene name	Primer sequence 5′–3′
*CAT* (CG6871)	CCAAGGGAGCTGGTGCTT
	ACGCCATCCTCAGTGTAGAA
*DREF* (CG5838)	GAAGCGGACCATTTCCAG
*WARTS* (CG12072)	GTTAGTGTGCGGAGCATTTC
	GCCGTCATCATCTTTGGCACA
*TTF* (CG18124)	ACTCAAGTCAATGGACTTTA
	TCTCAGAGTTCAGAGCACCCA
*TFB2* (CG3910)	TTCGGCGCCGTGGGCTCCTATC
	TGTATATGGGTGGGGCATG
*HIPPO* (CG11228)	CCTCTTCGGCAGCATCTC
	CCGAATCGGAGTTGATTACCATA
*OSA* (CG7467)	CCCTGTCCCTGTCTTCTCAC
	GATGGAACACCGGTAACCAC
*MOIRA* (CG18740)	CGACAAGGACGATGAAGAGG
	CGCTGATGATGATGTGGAAC
*P53* (CG33336)	CGCCCAAGTCTCTTTGGATGTACTCG
	CTTGAAGGCCAGGGTCTGGCGCGTG
*RP49* (CG7939)	CGATATGCCAAGCTAAAGCA
	GGGCGATCTCAGCACAGTAT

### Western blot

Cells (10^6^) were harvested, and total protein was extracted with 200 µL lysis buffer (20 mM Tris-HCl pH 8.0, 2 mM MgCl_2_, 0.2 M sucrose). Then, protein (20 µg) was resolved by 13% SDS-PAGE and transferred to a PVDF membrane (Millipore, Billerica, MA, USA) using wet electro-transfer. CAT, DREF, and endogenous control β-actin were blotted with corresponding antibodies^26,27^.

### ChIP-qPCR

Chromatin immunoprecipitation (ChIP) was performed using the iDeal ChIP-seq kit (Diagenode, Denville, NJ, USA) according to the manufacturer’s instruction with minor modifications. Briefly, 2 × 10^6^ PH_3_-treated or untreated cells were harvested in PBS and cross-linked with formaldehyde (1%) for 2 min. After sonication, chromatin from 1 × 10^6^ cells was subjected to ChIP with Pol II antibody (ab5408; Abcam, Cambridge, MA, USA). For ChIP-qPCR assays, four pairs of primers were used to amplify fragments at different distances downstream from the *DREF* transcription start site (TSS) (Table 3). Pol II enrichment was normalised to the constitutively active glyceraldehyde-3-phosphate dehydrogenase promoter region using the *Drosophila* Positive Control Primer Set Gapdh1 (Active Motif, Carlsbad, CA, USA).

**Table 3:** Primers for chromatin immunoprecipitation (ChIP)-qPCR.

Primer name	Sequence 5′–3′
DREF-0-ChIP-S	CAAACAAGAAGATCCCAATC
DREF-0-ChIP-R	TCCAAAGTAGCGCCAGTA
DREF-250-ChIP-S	CATCTCCAGCACCGACAC
DREF-250-ChIP-R	AATGAACTCCAGTTTGACCC
DREF-500-ChIP-S	AACCACGATAACGCTTCCG
DREF-500-ChIP-R	CGCTCCTCCTCCTCTACCA
DREF-1000-ChIP-S	CGCTTCCTTAGCATCTTC
DREF-1000-ChIP-R	CCTCTTCCTCGTCGTAGTT

### RNAi in S2 cells

RNAi was performed according to methods described by Fernández-Moreno *et al*. and Sawado *et al*.^23,31^. To make double-stranded RNAs, plasmid templates containing the corresponding target sequences were first constructed. For DREF, a fragment ranging from nucleotide positions 720–1,321 flanked by T7 promoter sequences at both ends was ligated into pUC19. For mock RNAi, a LacZ sequence, a fragment ranging from nucleotide positions 2,528–348 of the pUC19 vector, flanked by T7 promoters was ligated to pUC19. These templates were *in vitro*-transcribed using the MEGAscript® RNAi kit (Ambion, Austin, TX, USA) according to the manufacturer’s protocol. Next, *in vitro*-transcribed dsRNA (100 µg) was purified. For dsRNA treatment, 1.5 µg of dsRNA per 10^5^ S2 cells was directly added to unsupplemented TC100 medium. After 1 h of incubation, medium was changed to complete medium. After 24 h, the cells were transfected with luciferase reporters; 48 h later, cells were collected for real-time PCR, western blot, or luciferase activity assays.

### Transient gene overexpression in S2 cells

The full open reading frame of the *D*. *melanogaster DREF* gene (NM_078805.4) was synthesised by Beijing Protein Innovation (Beijing, China) and ligated into the pAc5.1/V5-His vector with *Xho*I and *Xba*I. The expression vector was then transfected into S2 cells together with the luciferase reporter for 48 h. The transfected cells were then treated with PH_3_ for another 4 h, followed by real-time PCR, western blot, or luciferase activity assays.

### Statistical analysis

All data were generated from at least three independent replicate experiments and processed with Microsoft Excel (Microsoft Corporation, Redmond, WA, USA). Final data are expressed as means ± SE. One-way ANOVA and LSD test were used to test for statistically significant differences at p < 0.05.

### Data availability

All data generated or analysed during this study are included in this published article.

## Electronic supplementary material

Supplementary Information
