# Supplementary material for: Phosphine inhibits transcription of the catalase gene through the DRE/DREF system in Drosophila melanogaster
Source: Sci Rep. 2017 Oct 10;7:12913. doi: 10.1038/s41598-017-13439-4 (PMC5635064; doi:10.1038/s41598-017-13439-4)
Supplement: Supplementary file 1 — Supplementary Information [file 41598_2017_13439_MOESM1_ESM.pdf]

Phosphine inhibits transcription of the catalase gene through the DRE/DREF system in *Drosophila melanogaster*

Tao Liu *, Li Li, Baishu Li, Guoping Zhan

Institute of Equipment Technology, Chinese Academy of Inspection and Quarantine, No. 241, Huixinxijie, Chaoyang District, Beijing, 100029, P.R. China

***Correspondence and requests for materials should be addressed to:** Tao Liu, Ph.D., Associate Professor. Tel.: +86-10-64969676; Fax: +86-10-64912751; Email: Liut@caiq.gov.cn

Supplementary Information

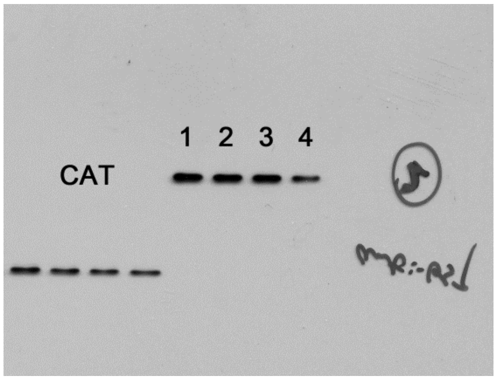

Supplementary Figure S1. Western blot against DmCAT in S2 cells treated with 14 μg/L PH_3 for 0 (lane 1), 1(lane 2), 2 (lane 3), or 4 h (lane 4).

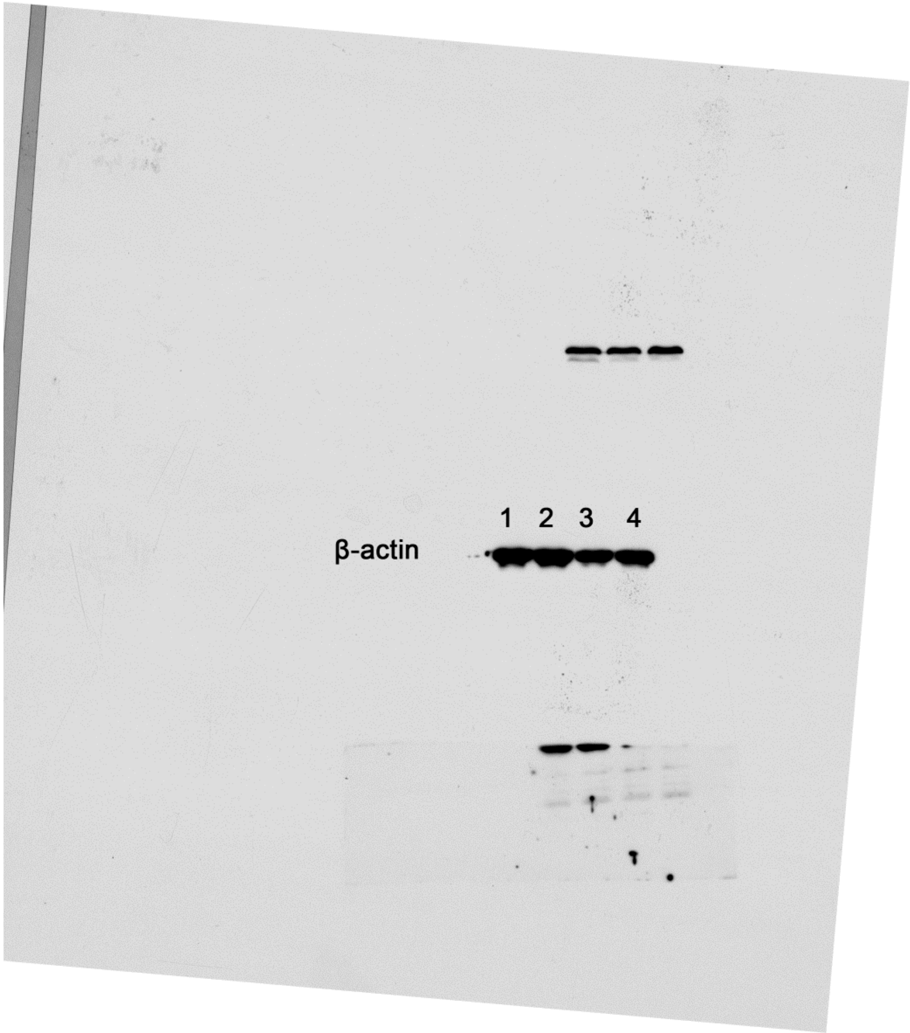

Supplementary Figure S2. Western blot against β-actin in S2 cells treated with 14 μg/L PH_3 for 0 (lane 1), 1(lane 2), 2 (lane 3), or 4 h (lane 4).

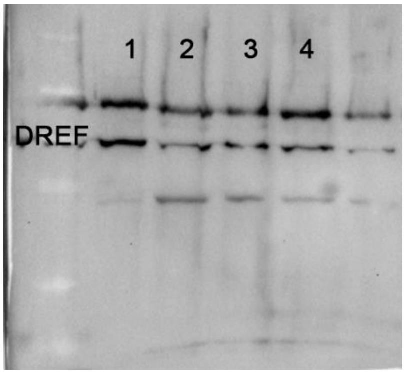

Supplementary Figure S3 Western blot analysis of *DREF* in S2 cells treated with 14 µg/L PH_3 for 0 (lane 1), 1(lane 2), 2 (lane 3), or 4 h (lane 4).

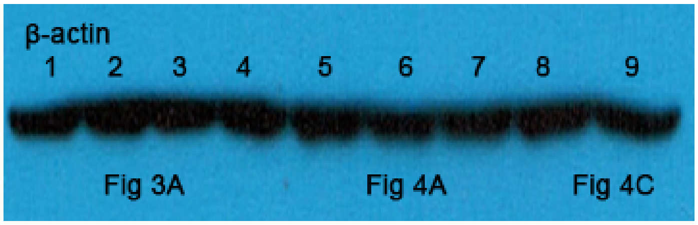

Supplementary Figure S4 Western blot against β-actin in S2 cells. Lane 1-4: Endogenous control for Fig. S3, Lane 5-7: Endogenous control for Fig. S5, Lane 8-9: Endogenous control for Fig. S6.

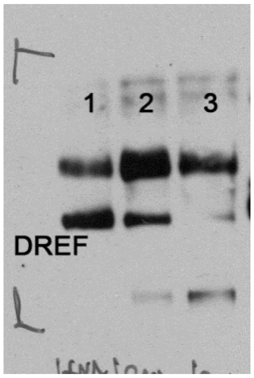

Supplementary Figure S5. Western blot validating the efficiency of *DREF* RNAi. Lane1: Control cells, Lane 2: Cells transformed with LacZ fragments, Lane 3: Cells transformed with DREF RNAi fragments.

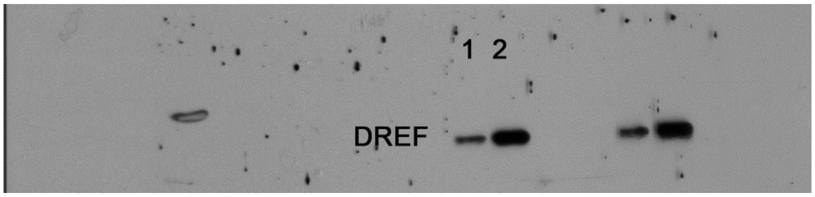

Supplementary Figure S6. Western blot validating the overexpression of DREF. Lane1: Control cells, Lane 2: Cells transformed with DREF overexpression vectors.

Supplementary Table S1. Promoter activities of wild-type and mutant DRE *DmCAT* promoters treated with or without PH_3.

Promoters	-1944 full length	-94	-94 M
Untreated	318.3 ± 30.7	426.7 ± 27.0	4.5 ± 0.6
PH_3	104.7 ± 10.5	116.9 ± 7.8	3.8 ± 0.5
